# Assessment of the Deterioration State of Post-Installed Bonded Anchors Using Ultrasonic

**DOI:** 10.3390/ma14082077

**Published:** 2021-04-20

**Authors:** Oliver Zeman, Michael Schwenn, Martin Granig, Konrad Bergmeister

**Affiliations:** Department of Civil Engineering and Natural Hazards, Institute of Structural Engineering, University of Natural Resources and Life Science (BOKU), 1190 Vienna, Austria; oliver.zeman@boku.ac.at (O.Z.); martin.granig@gmx.net (M.G.); konrad.bergmeister@boku.ac.at (K.B.)

**Keywords:** assessment of bonded anchors, loading history of anchors, ultrasonic

## Abstract

The assessment of already installed anchorages for a possible exceeding of the service load level is a question that is gaining more and more importance, especially in building maintenance. Bonded anchors are of particular interest here, as the detection of a capacity reduction or load exceedance can cause damage to the concrete-bonded mortar behavior. This article investigates the extent to which ultrasonic methods can be used to make a prediction about the condition of anchorages in concrete and about their load history. A promising innovative assessment method has been developed. The challenges in carrying out the experimental investigations are the arrangement of the transducers, the design of the test set-up and the applicability of direct, indirect or semidirect ultrasonic transmission. The experimental investigations carried out on a test concrete mix and a bonded anchor system show that damage to the concrete structure can be detected by means of ultrasound. The results indicate the formation of cracks and therefore a weakening of the response determined by means of direct, indirect and semidirect ultrasonic transmission. However, for application under non-laboratory conditions and on anchors with unknown load history, the calibration with a reference anchor and the identification of the maximum load is required. This enables a referencing of the other loaded anchors to the unloaded conditions and allows an estimation of the load history of individual anchors.

## 1. Introduction

Fastening technology has developed into a considerable and demanding technical discipline. The requirements for the various anchoring systems are becoming more complex and more extensive as the fields of application expand. In addition to the common destructive test methods for concrete, non-destructive material testing methods are increasingly requested for the assessment of already installed fastening systems. The non-destructive detection of the condition of the component enables the determination of the externally non-visible defects and damages in the concrete structure. Within this contribution the question is discussed, how ultrasound techniques can be applied for the assessment of already installed and loaded post-installed bonded anchors. The main question is if after an extreme loading situation, or within a quality insurance verification after the maximum designed lifetime the anchor is still in a safe status. By means of ultrasound characterization processes it is suggested to be able to assess the deterioration process and the crack development process of single bonded fasteners respectively. It is the main idea to test these non-destructive measurements in a first step on a bonded system due to the working principle over the whole load transfer zone. In contradiction to post-installed mechanical fasteners, the load transmission and hence the ultrasound transmission area are known for bonded fasteners with a higher certainty. As a result of this, it shall be possible to determine deteriorations or influences caused by the crack formations in the contact zone between bonded anchor and concrete specimen.

Research conducted by Hofmann and Laumann [1] show that the identification of the actual embedment depths is possible for post-installed mechanical fasteners with embedment depths up to approximately 100 mm. This shown research focuses on the determination of the unknown anchor length of already installed anchorages. The evaluation, if the anchorage shows any characteristics of a status where the design value already has been reached, was not the focus. Additionally, research was carried out on mechanical anchors in [2] and as the basis research for this contribution in [3] on chemical anchorages.

In a more advanced field of research, Wolf, Pirskawetz and Zang [4] have shown that the detection of cracks in reinforced concrete beams can be carried out using ultrasound. The development of the sound propagation time as a function of loading cycles and unloading cycles was demonstrated on a reinforced concrete structure. Feng, Zhao and Qiu show in [5] how damage imaging in mesoscale concrete can be performed by using the time-reversal method (TRM) combined with wavelet analysis. Casrellano et al. show in [6] the differences between linear and nonlinear ultrasonic techniques for detecting and monitoring damages in concrete. They used the nonlinear sideband peak count (SPC) technique for revealing the stress-induced damage corresponding to each load step. In comparison to linear ultrasonic parameters, the nonlinear ultrasonic parameter SPC-I appeared to be more sensitive to the variations of the internal material structures during both loading and unloading phases. [6]

The research provided within this contribution presents results of a tested bonded anchor system in a concrete slab under various loading situations (loaded in percentage of the ultimate load/unloaded after loading/ultimate load). Ultrasound measurements on different positions (direct, indirect and semidirect positioning) and evaluation processes have been used and are reported. Hence as a result a method will be suggested, how the deterioration state of an already installed bonded anchor system could be determined.

## 2. Materials and Methods

### 2.1. Ultrasound Measurements

The ultrasonic measurement method has traditionally been used for the quality control of materials and is very effective in these fields as shown exemplarily in [7,8]. The characterization of concrete as a multiphase, multiscale material was made by Kim, B.-C. and Kim, J.-Y. [9].

For the experiments presented the ultrasound device Pundit Lab+ (No. 326, Proceq, Schwerzenbach, Switzerland) is used. The portable device from the Swiss company Proceq is especially suitable for tests on concrete. The ultrasonic pulse velocity is determined via the signal propagation time t_1_ and the path length of the signal to be penetrated through the component. The waveform is displayed directly on the measuring device, enabling a first rough analysis after the measurement.

**Figure 1:**
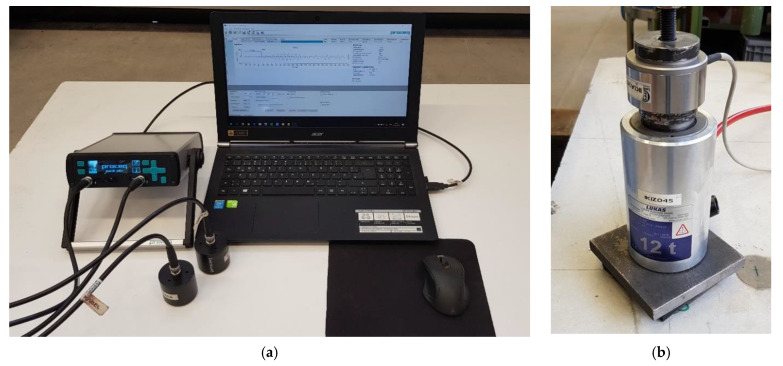
Overview of ultrasonic measurement configurations: (**a**) used ultrasound device Pundit Lab+; (**b**) test setup for load application.

Figure 1a shows the used Pundit Lab+ ultrasonic device including the corresponding Punditlink (version2.4.4, Proceq, Schwerzenbach, Switzerland) measurement software. The ultrasound device is connected to two transducers, which are used as transmitters and receivers of the signal wave. Different transducers can be used for measurements in the frequency range 24–500 kHz. The excitation pulse can be adjusted on the device, whereby the pulse voltage is in the range of 125–500 V. Furthermore, the received ultrasonic signal can be amplified, whereby the amplification bandwidth ranges from single to 1000-fold amplification.

The resolution of the signal is shown in the waveform display on the abscissa as time in μs, whereby it can be set in which time period signals are emitted or how long a signal is emitted by the measuring device. On the ordinate of the waveform display, the amplitude, i.e., the deflection of the measured echo, is indicated. This is unitless but is provided as a percentage value. The entire representation of the waveform corresponds to an A—image since the measurement is made at a point.

In order to be able to carry out measurements in the different frequency ranges, different transducers were used as listed in Table 1. Each pair of transducers is assigned its own frequency. Due to this tuning, the transducers also differ in their geometric shape. The following five pairs of transducers are used for the tests carried out: longitudinal wave transducers: 54 kHz, 54 kHz (exponential transducer), 150 kHz and 250 kHz and transverse wave transducer of 250 kHz.

**Figure 2:**
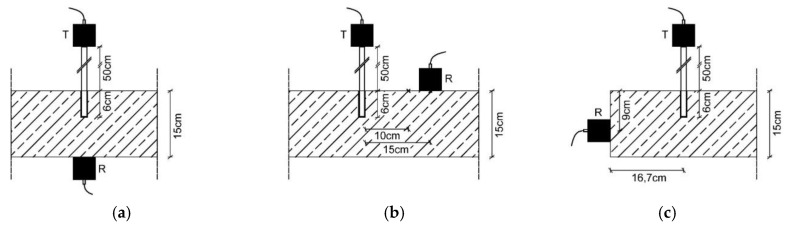
Overview of ultrasonic measurement configurations (*T—*transmitter; *R—*receiver). (**a**) direct transmission; (**b**) indirect transmission; (**c**) semidirect transmission.

The tests were carried out using direct, indirect and semidirect sound transmission, see Figure 2 for the schematic test setup with the used specification in the tests. In Figure 2 *T* denominates the transmitter and *R* denominates the receiver. In terms of physical principle, direct transmission is preferable to the others. In practice, however, the opposite side of a mounting surface (wall, ceiling panel, etc.) is rarely freely accessible. Therefore, a verification of the direct method with semidirect or indirect sound transmission is appropriate.

The measuring method used (in particular direct sound transmission) requires a structure that presses the probes with uniform pressure against the underside of the component.

Theoretically, the probes can also be pressed against the underside of the component by hand. However, this method ensures inaccurate and not replicable measurements. Therefore, a designed-in-house wooden construction is used on which all probes can be clamped.

**Figure 3:**
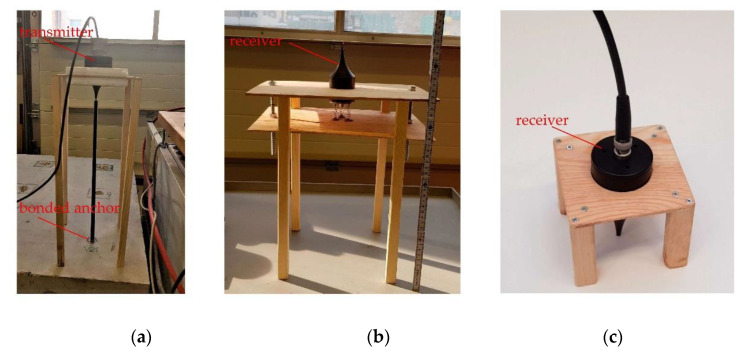
Test setups for different ultrasonic measurement configurations: (**a**) test setup for all types of transmission, top side; (**b**) test setup for direct transmission, bottom side; (**c**) test setup for indirect transmission, top side.

Wood was deliberately chosen as the material for this fixture in order to keep the disturbing influence on the ultrasound wave as small as possible. With a constant contact pressure of 1 kg, the transducers are equally loaded. In addition to making it easier for the transducers to stand alone and stable in the wooden frame, repeatable and stable measurements can be made, see Figure 3.

### 2.2. Loading Device

As a loading device, a hydraulic cylinder with a corresponding hydraulic aggregate was used, see Figure 1b. This enables one to load the bonded anchor up to certain load steps and hold the load level for a certain time.

Two rubber soundproofing panels are placed directly on the surface of the concrete slab and a steel plate of similar geometric dimensions is placed on top of them. This ensures full-surface pressure distribution and decupling of the contact area between cylinder and concrete. This is threaded into the threaded rod through a central opening before loading. In addition, the load was measured by means of a load cell positioned centrically in the axis of the threaded rod of the bonded anchor.

### 2.3. Materials

#### 2.3.1. Concrete Specimen

According to the scale separation principle, the maximum grain size that occurs specifies the wavelength of the ultrasonic waves. All tests of this contribution have been performed with concrete slabs of the same size, manufacturing date and mixture. This is due to the decision that in a first step influences from different concrete mixtures shall be excluded to focus on the behavior of the deterioration process of the bonded fastener under load itself. The considered concrete specimens had a size of length *l* = 100 cm, width *b* = 100 cm and height *h* = 15 cm and a nominal strength class of C20/25 in accordance with EN 206 [11]. The concrete specimens were put under laboratory conditions and an actual concrete compressive strength at time of testing of *f_c,cube150,test_* = 25.4 N/mm^2^ measured on cubes. The mixture of the concrete consists of round aggregates with a maximum size of *d_max_* = 16 mm with resistances to fragmentation of a Los Angeles abrasion value of 28 and an impact value of 21. Used cement type was CEM I 32.5 R in accordance with EN 197 [12], the water cement ratios was approximately *w/z* = 0.7. Figure 1 shows the grading curve of the used aggregates of the concrete mixture. The grading curve of the used aggregates was within the so called favorable area in accordance with ÖNORM B 4710, which characterizes the range in which the gradation of the aggregates is at its optimum for the internal structure of the material properties.

#### 2.3.2. Bonded Anchor System

**Figure 4:**
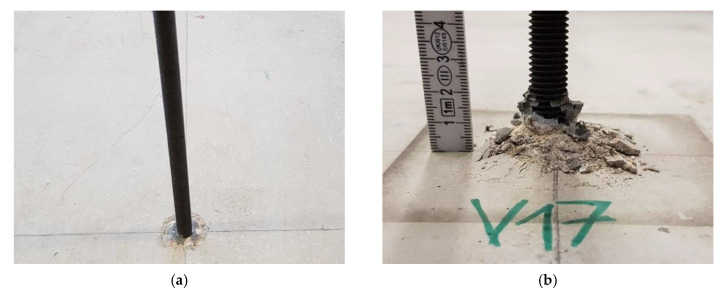
Overview of the bonded anchor system used in the test series. (**a**) installation of anchor system; (**b**) typical failure mode.

Post-installed bonded anchors are acting by a load transmission over the whole embedment depth of the anchorage. In the considered test schedule a two-component epoxy resin mortar was used. This mortar consists of epoxy resin as binding material, which hardens through polyaddition, hardening material and fillers. The mortar is intended to be used in non-cracked and cracked concrete for applications with an embedment depth from 60 to 600 mm with various steel elements as exemplarily reinforcement bars or threaded rods of the diameter M8 to M30. This mortar consists of an injection cartridge and an appropriate static mixer and is intended to be used for structural applications in reinforced or nonreinforced concrete of strength classes C20/25 to C50/60 in accordance with EN 206 [11]. In case of the presented research, standard threaded rods of nominal diameter M12 of strength class 12.9 and a length of *l* = 50 cm have been used (tensile strength *f_u_* = 1200 N/mm^2^ and yield strength *f_y_* = 1080 N/mm^2^), see Figure 4a. To carry out the subsequent ultrasonic measurements, the threaded rod ends must be ground flat to create as smooth and regular a contact surface as possible for the measurement. The embedment depth was set to *h_ef_* = 60 mm for all considered tests to avoid steel failure of the threaded rod in testing. The minimum thickness of *h_min_* = 10 cm for size M12 was fulfilled with the height of the concrete member of *h* = 15 cm. In the considered tests, the drill holes were hammer drilled with a four-cutting edge driller of *d_cut_* = 14.35 mm. The drilled holes were cleaned by blowing in of compressed, oil-free air (2×), brushing by a cleaning brush (2×) and blowing in of compressed, oil-free air (2×). Tests have been performed after a minimum considered curing time of *t_cure_* ≥ 24 h. A typical failure mode after conduction of the tests is shown in Figure 4b.

### 2.4. Overview of Test Program

The tests reported here are listed in Table 2. The composite anchors were measured at different load levels. This means that measurements are taken after a defined load has been applied. Measurements under continuous increasing load application were not carried out. The load steps were as follows: 0 kN, 1 kN, 20 kN, 40 kN and 60 kN. After each load level, the anchor was unloaded again to 1 kN (reference load level) and an ultrasonic measurement was taken again.

The load levels were determined in such a way that the ultimate loads of the used fastening system are approximately 70–95 kN. Therefore, the last load level of 60 kN is still sufficiently far away from failure, but already significantly above the service load level.

In the zero measurements, only the unloaded threaded rod is sounded. Slight deviations from each other result from the gluing process in the drill hole. Small air inclusions in the adhesive can interfere with the full bonding of the threaded rod and cause a deviation of the transmission signal.

The 1 kN measurement is taken after all the test equipment has been set up and serves as a reference value for the other load level measurements and as a reference value for the unloaded state.

**Figure 5:**
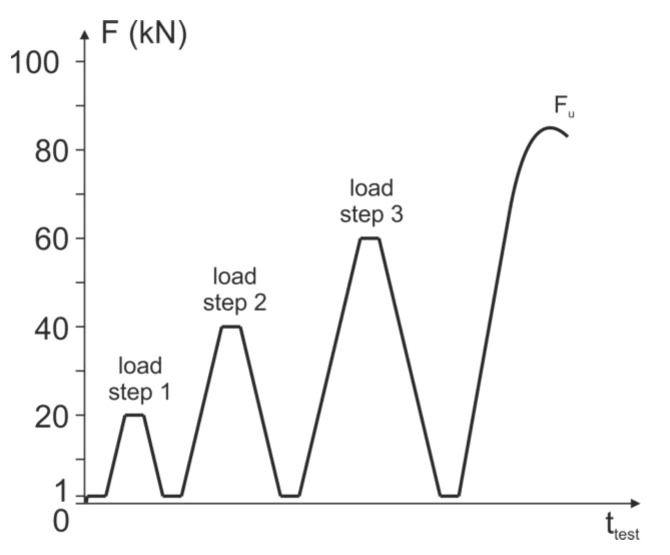
Schematic loading procedure for the conducted investigations, see [2] for additional information.

The other load levels are, as mentioned, 20 kN and 40 kN. At the last load level of 60 kN, acoustic signals indicate that first cracks are appearing within the concrete structure. Visually recognizable, superficial damage could not be detected. After the last measurement, the threaded rod is loaded until failure. The schematic loading procedure is depicted in Figure 5.

## 3. Results

The presentation of the results is based on the one hand on the signal propagation times and on the other hand on the detected waveforms. For comparison with direct transmission, where a larger number of tests are available, the tests with indirect and semidirect transmission are also shown.

### 3.1. Direct Transmission

#### 3.1.1. Results Based on Signal Propagation Time

**Figure 6:**
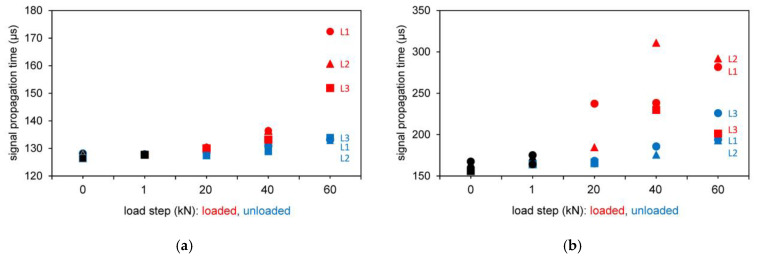

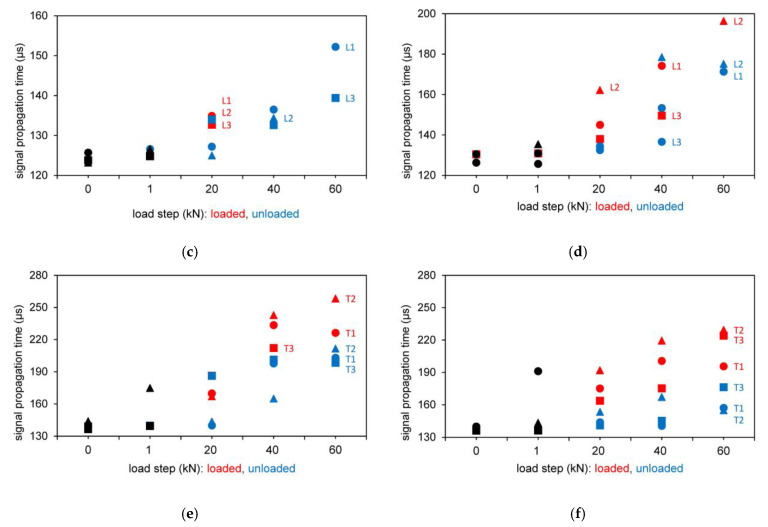
Results based on signal propagation time for different longitudinal wave transducers (54 kHz, 54 kHz (exponential transducer), 150 kHz and 250 kHz) and transverse wave transducer (250 kHz with different configuration) for direct transmission. (**a**) 54 kHz; (**b**) 54 kHz Exp.; (**c**) 150 kHz; (**d**) 250 kHz; (**e**) 250 kHz transverse orthogonal; (**f**) 250 kHz transverse parallel

The test results of the signal propagation time for the different load steps (loaded and unloaded) for direct transmission are shown in Figure 6. In the diagrams, the test series L1, L2 and L3 resp. T1, T2 and T3 (different data point shape) are shown in the initial loading step from 0 to 1 kN (black color) and in loaded (red color) and unloaded (blue color) situation for various loading steps.

The loaded (red) and unloaded (blue) measured values plotted in Figure 6 show that the increases in the signal propagation time are more noticeable in the loaded state. The relieved measured values, on the other hand, scatter less strongly in the comparison of the individual tests and therefore have a better comparability.

The first assumption suggests an increase in the signal propagation time with increasing load level. This is probably due to the fact that the ultrasonic wave can only penetrate the concrete structure more slowly as the pull-out load is approached. Both loaded and unloaded measurements show this phenomenon. The data from the shear wave transducer confirm the trend.

#### 3.1.2. Results Based on the Wave Area

In order to not only rely on the signal propagation times, an additional method is attempted for the evaluation. Therefore, the wave shapes are compared with each other. In order to enable a quantitatively comparison of different shapes of the waves, the total area of each wave was calculated. As a result of an unknown root function, the method of numerical integration was used.

**Figure 7:**
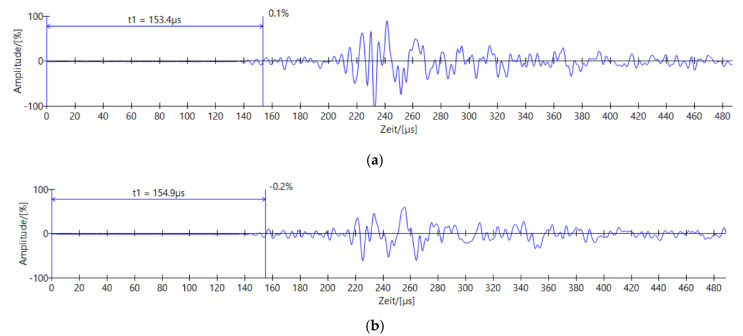
Examples of ultrasonic signals after different load steps, the area is defined between the abszissa and the wave, the procedure is described in [2]. (**a**) example of ultrasonic respond after unloading the 60 kN load step; (**b**) example of ultrasonic respond after unloading the 60 kN load step.

Figure 7 shows examples of waves, which were the computation base, as example Figure 7a shows the load step of 20 kN, whereas Figure 7b depicts a loading of 60 kN.

**Figure 8:**
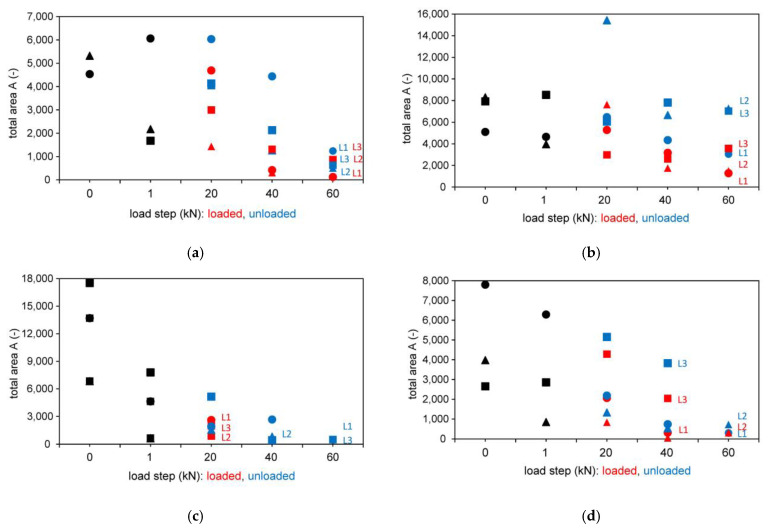

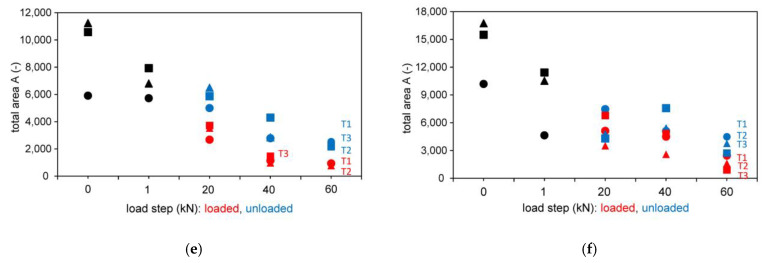
Results based on wave shape for different longitudinal wave transducers (54 kHz, 54 kHz (exponential transducer), 150 kHz and 250 kHz) and transverse wave transducer (250 kHz with different configuration) for direct transmission. (**a**) 54 kHz; (**b**) 54 kHz Exp.; (**c**) 150 kHz; (**d**) 250 kHz; (**e**) 250 kHz transverse orthogonal; (**f**) 250 kHz transverse parallel

In Figure 8, the abscissa thus shows the load levels, the ordinate the area contents plotted in absolute units for the tests with direct transmission. In the diagrams, the test series L1, L2 and L3 resp. T1, T2 and T3 (different data point shape) are shown in the initial loading step from 0 to 1 kN (black color) and in loaded (red color) and unloaded (blue color) situation for various loading steps.

The areas of the waveforms seem to become smaller with increasing load level. Due to the damping of the wave and the losses from the sound attenuation, information is lost through the structural component. This means that changes in the concrete structure cause these strong deviations. The values of the surface areas themselves, plotted on the ordinates of the diagrams, are not comparable with each other, as the measurements are carried out with the probes in different signal amplifications, see Section 2.1. The wave images also do not allow an estimation of the size and extent of the damage. Therefore, only changes in the structure itself can be determined, however no quantification is possible.

### 3.2. Indirect and Semidirect Transmission

In a further step, indirect and semidirect transmission was applied. Only selected results are provided here, but the results are similar for all used transducers.

#### 3.2.1. Results Based on Signal Propagation Time

**Figure 9:**
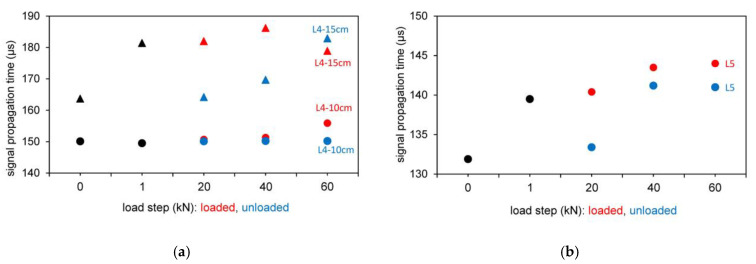
Results based on signal propagation time for different longitudinal wave transducers 54 kHz (exponential transducer) and 250 kHz. (**a**) 54 kHz Exp.; (**b**) 250 kHz.

The test results of the signal propagation time for the different load steps (loaded and unloaded) are shown in Figure 9 for the tests with indirect and semidirect transmission. In Figure 9, the test series L4 and L5 are shown in the initial loading step from 0 to 1 kN (black color) and in loaded (red color) and unloaded (blue color) situation for various loading steps.

#### 3.2.2. Results Based on the Wave Shape

**Figure 10:**
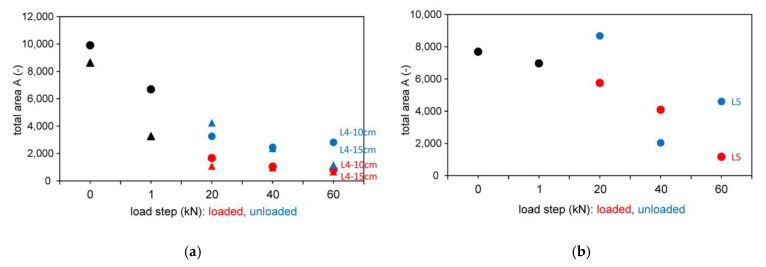
Results based on wave shape for different longitudinal wave transducers 54 kHz (exponential transducer) and 250 kHz. (**a**) 54 kHz Exp.; (**b**) 250 kHz.

In Figure 10, the tests with indirect and semidirect transmission are shown evaluated based on the wave shape. In Figure 10, the test series L4 and L5 are shown in the initial loading step from 0 to 1 kN (black color) and in loaded (red color) and unloaded (blue color) situation for various loading steps.

## 4. Discussion and Conclusions

The aim of this work show how an ultrasonic method can be used to make an evaluation about the condition of already installed bonded anchors in concrete. Different measuring methods are to show to what extent changes in the running time and the wave processes are in direct correlation with the fracture pattern that is forming within the concrete. The goal of successfully applying non-destructive testing by means of ultrasound examinations can be seen as achieved in principle. A damage pattern can definitely be determined, whereas no quantification can be made.

**Figure 11:**
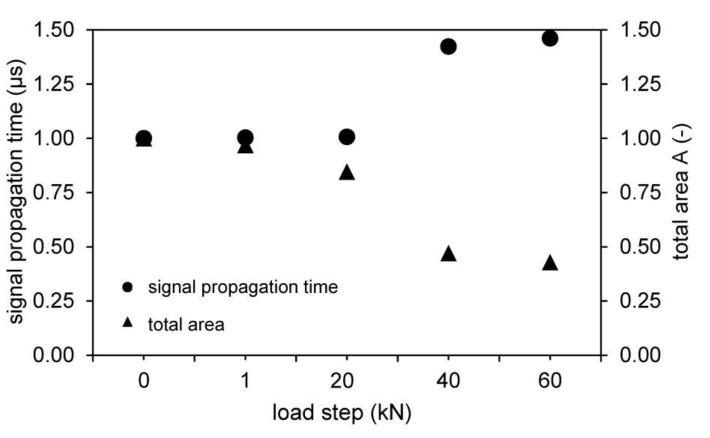
Schematically evaluation based on signal propagation time and wave shape for direct, indirect and semidirect transmission.

It is explicitly stated that a change in the running time is directly correlated to the developing fracture pattern as it is shown in Figure 6 and Figure 8, Figure 9 and Figure 10. The signal propagation times of the passing wave become longer with increasing load level. While there are only minor changes after the zero measurements, the 1 kN and unloaded 20 kN load level measurements, the curve rises for the loaded and unloaded 40 kN and 60 kN measurements. Figure 11 shows exemplarily the behavior of one of the tests measured with the 250 kHz shear wave transducer. While the left ordinate shows the signal propagation times in μs, the vertical right axis shows the area of the waveforms (no unit). In addition to the change in signal propagation time, it can also be demonstrated that the wave shapes decrease with increasing load on the anchoring elements, which results in smaller calculated surface areas of the waveforms. The trend that can be observed in the shown tests confirms damage in the overall structure.

The use of the experimental results shown here in reality is possible in principle. At first glance, the realization appears to be difficult. It could be impressively shown that a statement about the load history of an anchor can be made from the measurements. However, the unloaded condition (i.e., the reference value) is known for the tested specimens.

In a real application of this method on installed anchors, where it is to be investigated whether they have already been under a load that has caused damage in the microstructure of the concrete, this zero state is, however, difficult to realize. One possibility would be the targeted application of a reference anchor, which is not loaded and can thus be used as a reference specimen. This reference anchor experiences the same environmental conditions and ageing processes, but always remains unloaded and allows the calibration of the other fasteners to the unloaded state later.

The validation of the tests carried out with indirect or semidirect sonication is particularly important. In general, the fastening substrate is not equally accessible from all sides. Therefore, direct sound transmission is usually not suitable.

Summarizing as a conclusion, the following conditions must be considered for an assessment of the load history of a bonded anchor

1. The anchor must be accessible and at least indirect through-sounding to the fixing substrate must be feasible;
2. The measurements should be validated on newly installed systems in the same substrate, in particular to exclude the effects of different aggregates, different cementitious matrix or present reinforcement;
3. An unloaded reference anchor of the same age as the anchors to be tested shall be available;
4. The ultimate load should be determined on a “victim anchor” to be able to evaluate the damage;
5. The assessment can be made on the basis of the signal propagation time and on the basis of the integrated area of the sound wave, which can be seen as assessment criteria.

This method presented in this article is certainly not to be regarded as already completely ready for final use. However, the fundamental applicability of this method has been properly proven by the research conducted. Further investigations and considerations on the technical implementation in practice should follow.

## Figures and Tables

**Table 1:** Overview of the used transducers and their typical applications according to information provided in [10].

Spectrum	Requirements for the Object of Study			Application
	Wavelength	Maximum Aggregate Size (mm)	Minimum Lateral Dimension (mm)	
Longitudinal wave transducers				
54 kHz	68.5	approx. 34	69	Concrete, wood, solid rock
				
150 kHz	24.7	approx. 12	25	Fine-grained materials, fireclay, solid rock
				
250 kHz	14.8	approx. 7	15	Fine-grained materials, fireclay, rockmass, especially small specimens
				
54 kHz Exp.	68.5	approx. 34	69	Concrete: rough and curved surfaces; wood, solid rock
				
Transverse wave transducer				
250 kHz	10.0	approx. 5	Larger than thickness of object	Determination of modulus of elasticity: concrete, wood, solid rock, special connection paste required

**Table 2:** Overview of test program with the bonded anchor system.

Test no. ^(1)^	Threaded Rod	Transducer (kHz)	Transmission	Ultimate Load (kN)
L1	M12 12.9	54, 54 (exp.), 150, 250	direct	68.2
L2	M12 12.9	54, 54 (exp.), 150, 250	direct	92.3
L3	M12 12.9	54, 54 (exp.), 150, 250	direct	92.0
L4	M12 12.9	54, 54 (exp.), 150, 250	indirect	88.1
L5	M12 12.9	54, 54 (exp.), 150, 250	semi direct	86.5
T1	M12 12.9	250	direct	74.3
T2	M12 12.9	250	direct	82.4
T3	M12 12.9	250	direct	87.0
T4	M12 12.9	250	indirect	85.2
T5	M12 12.9	250	semi direct	94.6

^(1)^ L—longitudinal wave transducers: 54 kHz, 54 kHz (exponential transducer), 150 kHz and 250 kHz; T—transverse wave transducer of 250 kHz.

## Data Availability

Data is not shared.
